# Supplementary material for: A maternally programmed intergenerational mechanism enables male offspring to make piRNAs from Y-linked precursor RNAs in Drosophila
Source: Nat Cell Biol. 2023 Sep 18;25(10):1495–505. doi: 10.1038/s41556-023-01227-4 (PMC10567549; doi:10.1038/s41556-023-01227-4)
Supplement: Supplementary file 1 — Reporting Summary [file 41556_2023_1227_MOESM1_ESM.pdf]

nature portfolio

Corresponding author(s): Yukiko Yamashita

Last updated by author(s): Jul 10, 2023

Reporting Summary

Nature Portfolio wishes to improve the reproducibility of the work that we publish. This form provides structure for consistency and transparency in reporting. For further information on Nature Portfolio policies, see our Editorial Policies and the Editorial Policy Checklist.

Statistics

For all statistical analyses, confirm that the following items are present in the figure legend, table legend, main text, or Methods section.

n/a	Confirmed	
☐	☒	The exact sample size (*n*) for each experimental group/condition, given as a discrete number and unit of measurement
☐	☒	A statement on whether measurements were taken from distinct samples or whether the same sample was measured repeatedly
☐	☒	The statistical test(s) used AND whether they are one- or two-sided<br>*Only common tests should be described solely by name; describe more complex techniques in the Methods section.*
☒	☐	A description of all covariates tested
☒	☐	A description of any assumptions or corrections, such as tests of normality and adjustment for multiple comparisons
☐	☒	A full description of the statistical parameters including central tendency (e.g. means) or other basic estimates (e.g. regression coefficient) AND variation (e.g. standard deviation) or associated estimates of uncertainty (e.g. confidence intervals)
☐	☒	For null hypothesis testing, the test statistic (e.g. *F*, *t*, *r*) with confidence intervals, effect sizes, degrees of freedom and *P* value noted<br>*Give P values as exact values whenever suitable.*
☒	☐	For Bayesian analysis, information on the choice of priors and Markov chain Monte Carlo settings
☒	☐	For hierarchical and complex designs, identification of the appropriate level for tests and full reporting of outcomes
☐	☒	Estimates of effect sizes (e.g. Cohen's *d*, Pearson's *r*), indicating how they were calculated

Our web collection on statistics for biologists contains articles on many of the points above.

Software and code

Policy information about availability of computer code

Data collection	Illumina NextSeq 550, Leica TCS SP8 confocal microscope
Data analysis	fastx toolkit (v0.0.14); bowtie2 (v2.2.0); STAR (v2.3.1); StringTie (v1.3.4); bowtie (v1.0.0); SAMtools (v1.0.0); DESeq2 (v1.18.1); Microsoft Excel 2013; ImageJ

For manuscripts utilizing custom algorithms or software that are central to the research but not yet described in published literature, software must be made available to editors and reviewers. We strongly encourage code deposition in a community repository (e.g. GitHub). See the Nature Portfolio guidelines for submitting code & software for further information.

Data

Policy information about availability of data

All manuscripts must include a data availability statement. This statement should provide the following information, where applicable:

- Accession codes, unique identifiers, or web links for publicly available datasets
- A description of any restrictions on data availability
- For clinical datasets or third party data, please ensure that the statement adheres to our policy

Sequencing data generated in this study have been deposited in the National Center for Biotechnology Information Short Read Archive database under accession code PRJNA879723 and are available at https://www.ncbi.nlm.nih.gov/bioproject/PRJNA879723/. Fly genome sequence and annotation (build dm6/BDGP6.22 release 98) used in this study were downloaded from Ensembl at ftp://ftp.ensembl.org/pub/release-98/fasta/drosophila_melanogaster/ and ftp://ftp.ensembl.org/

pub/release-98/gtf/drosophila_melanogaster/; fly rRNA sequences were downloaded from SILVA rRNA database at https://www.arb-silva.de/. Code used in this work is deposited at https://github.com/ildargv/Venkei_et_al_2023

Human research participants

Policy information about studies involving human research participants and Sex and Gender in Research.

Reporting on sex and gender	N/A
Population characteristics	N/A
Recruitment	N/A
Ethics oversight	N/A

Note that full information on the approval of the study protocol must also be provided in the manuscript.

Field-specific reporting

Please select the one below that is the best fit for your research. If you are not sure, read the appropriate sections before making your selection.

☒ Life sciences ☐ Behavioural & social sciences ☐ Ecological, evolutionary & environmental sciences

For a reference copy of the document with all sections, see nature.com/documents/nr-reporting-summary-flat.pdf

Life sciences study design

All studies must disclose on these points even when the disclosure is negative.

Sample size	No statistical method was used to determine the sample size. For all biological samples, the maximum possible sample size (n = 3–90) was chosen for each type of data ensuring that variability arising from all accountable sources was incorporated in the analyses (day of data collection, reagent lots, experimenter).
Data exclusions	No data were excluded from the analyses.
Replication	All data were collected during independent trials (n = 3) conducted on separate days. All attempts at replication were successful. When using several types of data for analyses, all possible permutations of samples were analyzed (e.g., 3 small RNA sequencing × 3 5' monophosphorylated RNA sequencing data sets produced 9 permutations). All attempts at replication were successful.
Randomization	This study did not involve treatment or exposure of animals to any agent. Instead, the goal of this work was to compare untreated wild-type/control flies and untreated mutant flies: all wild-type animals were compared to all mutant animals. Therefore, randomization is not relevant to this study.
Blinding	Blinding is not relevant to this study. Blinding was not performed during data collection, because methods used for data acquisition (smFISH, Western blotting, RT-qPCR, high-throughput sequencing) are not influenced by the experimenter's knowledge of the fly genotype. Blinding was not performed during data analyses, because analyses were performed with the same automated algorithms and programming code. During analyses, wild-type control and mutant data sets are also easily identified and are directly compared one to another.

Reporting for specific materials, systems and methods

We require information from authors about some types of materials, experimental systems and methods used in many studies. Here, indicate whether each material, system or method listed is relevant to your study. If you are not sure if a list item applies to your research, read the appropriate section before selecting a response.

Materials & experimental systems

n/a	Involved in the study	
☐	☒	Antibodies
☒	☐	Eukaryotic cell lines
☒	☐	Palaeontology and archaeology
☐	☒	Animals and other organisms
☒	☐	Clinical data
☒	☐	Dual use research of concern

Methods

n/a	Involved in the study	
☒	☐	ChIP-seq
☒	☐	Flow cytometry
☒	☐	MRI-based neuroimaging

Antibodies

Antibodies used	Mouse anti–α-Tubulin (clone 4.3; 1:3,000)(Walsh 1984) was obtained from the Developmental Studies Hybridoma Bank. The generation of polyclonal anti-Ste antibody (used at 1:10,000) was outsourced to Covance (Princeton, NJ) and was produced by immunizing guinea pigs with KLH conjugated Ac-KPVIDSSSGLLYGDEKKWC (53-70aa of Ste); horseradish peroxidase (HRP)-conjugated goat anti-mouse IgG (#115-035-003; 1:10,000; Jackson ImmunoResearch Laboratories), and anti-guinea pig IgG (#106-035-003; 1:10,000; Jackson ImmunoResearch Laboratories).
Validation	https://www.jacksonimmuno.com/catalog/products/106-035-003 ; https://www.jacksonimmuno.com/catalog/products/115-035-003

Animals and other research organisms

Policy information about studies involving animals; ARRIVE guidelines recommended for reporting animal research, and Sex and Gender in Research

Laboratory animals	Drosophila melanogaster w1118 (0–7 day old). The following lines were used: C(1)RM/C(X:Y)y1f1w1, armi1, armi72.1, aubHN2, aubQC42, zucEY11457, Df(2L)BSC323, nos-gal4:VP16, bam-gal4:VP16, UAS-flag3-myc6-ago3, UAS-gfp-aub, UAS-armi-gfp, UAS-dpp, TRIP.GL00254, TRIP.GL00076, TRIP.HMC02938, TRIP.HMS00373, TRIP.GL00111, UAS-gfp-Ste (SteXh:CG42398).
Wild animals	The study did not involve wild animals.
Reporting on sex	Findings specifically apply to male progeny from female X male crosses.
Field-collected samples	The study did not involve field-collected samples.
Ethics oversight	Work on Drosophila melanogaster does not require ethical oversight or experimental approval.

Note that full information on the approval of the study protocol must also be provided in the manuscript.
