# Supplementary figures and images for: A maternally programmed intergenerational mechanism enables male offspring to make piRNAs from Y-linked precursor RNAs in Drosophila
Source: Nat Cell Biol. 2023 Sep 18;25(10):1495–505. doi: 10.1038/s41556-023-01227-4 (PMC10567549; doi:10.1038/s41556-023-01227-4)

Fig. 3i

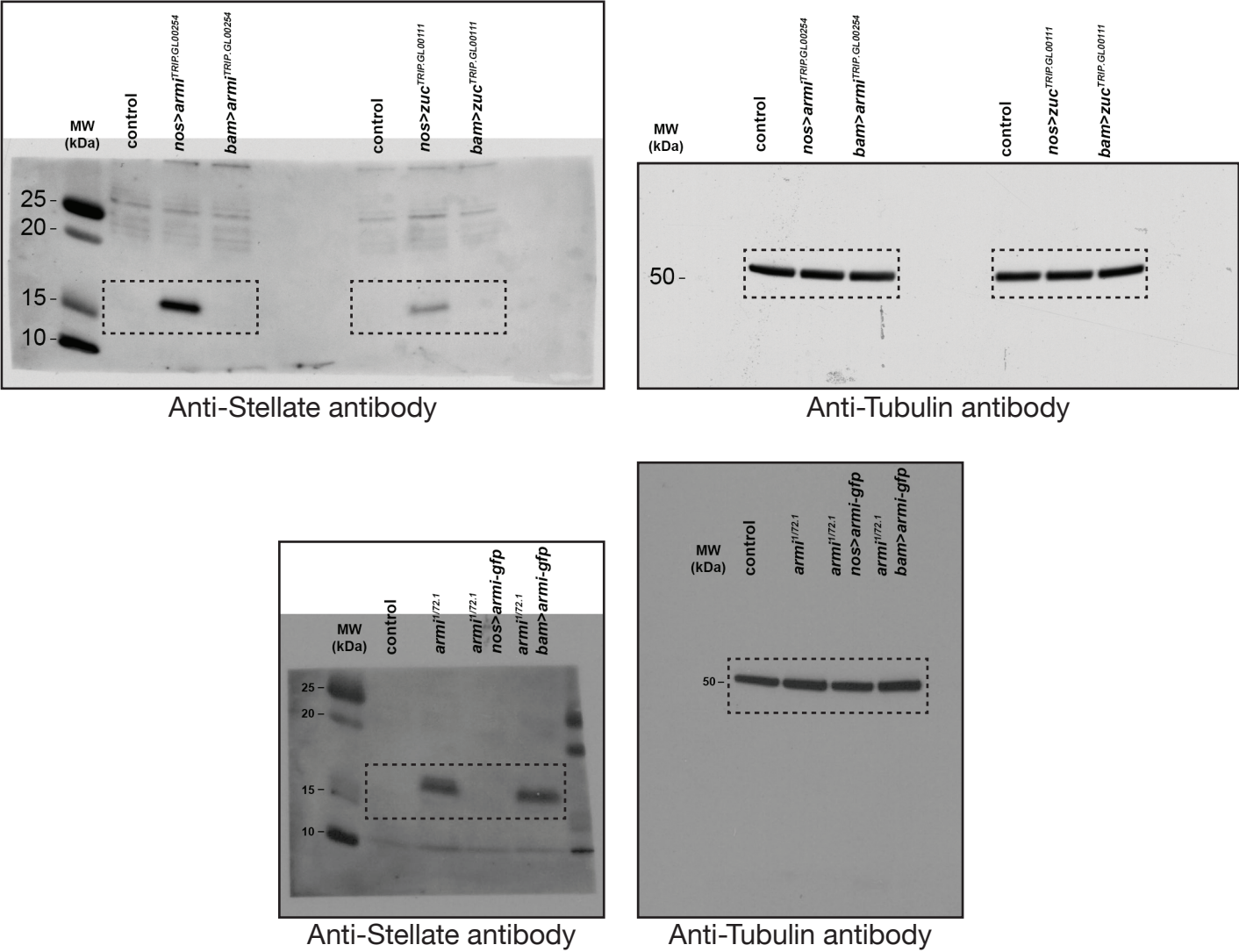

Extended Data Fig. 7

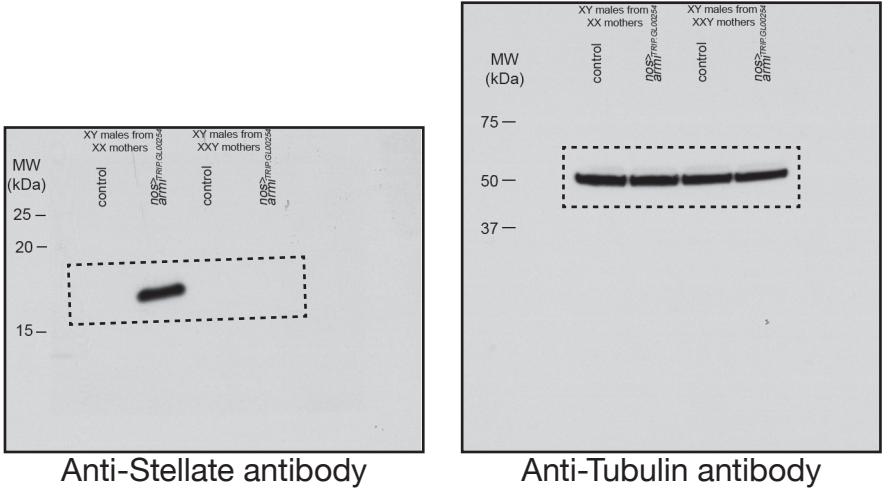

Supplement: Supplementary file 5 — Uncropped scans of western blotting data for Fig. 3i and Extended Data Fig. 7. [file 41556_2023_1227_MOESM5_ESM.pdf]
